# The interplay between prematurity, maternal stress and children’s intelligence quotient at age 11: A longitudinal study

**DOI:** 10.1038/s41598-018-36465-2

**Published:** 2019-01-24

**Authors:** Hélène Turpin, Sébastien Urben, François Ansermet, Ayala Borghini, Micah M. Murray, Carole Müller-Nix

**Affiliations:** 1 University Service of Child and Adolescent Psychiatry, University Hospital of Lausanne, Lausanne, Switzerland; 2 Department of Child and Adolescent Psychiatry, University Hospital, Geneva, Switzerland; 3 The LINE (Laboratory for Investigative Neurophysiology), Radiology Department and Department of Clinical Neurosciences, University Hospital Center and University of Lausanne, Lausanne, Switzerland; 4 The EEG Brain Mapping Core, Center for Biomedical Imaging (CIBM), University Hospital Center and University of Lausanne, Lausanne, Switzerland; 5 Department of Ophthalmology, University of Lausanne, Fondation Asile des Aveugles, Lausanne, Switzerland; 6 Department of Hearing and Speech Sciences, Vanderbilt University, Nashville, TN USA

## Abstract

Very premature children (<33 weeks of gestational age (GA)) experience greater academic difficulties and have lower, though normal-range, intelligence quotients (IQs) versus their full-term peers. These differences are often attributed to GA or familial socio-economic status (SES). However, additional factors are increasingly recognized as likely contributors. Parental stress after a child’s premature birth can present as post-traumatic stress disorder (PTSD) symptoms and can in turn reinforce difficulties in parent-child interaction pattern. Following a longitudinal design, we studied the interplay between a premature child’s perinatal history and maternal PTSD symptoms on intelligence abilities assessed at 11 years of age. Thirty-three very preterm and 21 full-term mother-children dyads partook in the study. Children’s perinatal risk was evaluated at hospital discharge, maternal PTSD symptoms were assessed when the children were 18 months old, and children’s IQ was measured at 11 years old. IQ was significantly lower for preterm than full-term children, without reliable influences from perinatal risk scores. However, lower maternal PTSD symptoms predicted higher IQ in preterm children. This preliminary study highlights the importance detecting maternal PTSD symptoms after a preterm birth and suggests interventions should target reducing maternal PTSD symptoms during early childhood to enhance very preterm children’s intelligence development.

## Introduction

Approximately 10% of babies are born prematurely (<37 weeks of gestation), and the number of survivors has increased due to medical, nursing and technological improvements in the neonatal intensive care unit (NICU)^1^. Preterm-born children, even those without severe neurological disabilities, present more difficulties than their full-term peers in academic achievement, persisting into early adolescence^2–6^. These difficulties can manifest as lower intelligence quotient (IQ) scores for preterm-born children than their full-term peers. The magnitude of this difference is reported to be approximately one standard deviation (i.e. roughly 10 points) lower than the average IQ score of full-term children. However, it is noteworthy that preterm children’s IQ scores nonetheless remain in the normal range^7^. Moreover, this difference persists into adulthood and exhibits an influence from the familial socio-economic status (SES)^8,9^.

Such notwithstanding, it is undeniable that the premature birth of a child is particularly difficult emotionally both for the child and the parents and can be a traumatic event^10^. Premature newborns’ development is immature, and they are physiologically unready for the extra-uterine environment^11^. Moreover, during the NICU stay, the sensory environment is highly atypical; there is separation from the mother, and painful and stressful medical care is often necessary^12–15^. These experiences have a widespread impact on later developmental outcomes, including in the endocrine system, brain microstructure, cognition and behavior^11,13,16–21^. Concerning the separation from the mother and the alteration of the sensory environment, a study on rat pups^22^ showed that the lack of tactile stimulation inhibits the secretion of growth hormones and activity of ornithine decarboxalase. In turn, these alterations affect tissue differentiation processes (e.g. brain development). The same study showed that human preterm born infants who benefited from tactile stimulation have better performance in cognitive and motor assessments than infants without tactile stimulation. Moreover, previous studies^23–25^ showed the beneficial effect of skin-to-skin care on preterm-born child’s development. Painful medical care also seems to impact the child’s IQ development. Grunau, *et al*.^15^ showed that the more often a preterm baby had skin-breaking procedures during the stay in NICU, the lower was the IQ score at 18 months of age. Studies in rats and humans^20,26^ showed that neonatal pain increases cell death in cortical areas and reduces white matter, and Peterson *et al*.^21^ showed that smaller cortical volume is associated to lower IQ scores. Moreover, Vinall *et al*.^27^ showed that the greater is the number of invasive procedures during the NICU stay, the lower is the fractional anisotropy value of the superior white matter, which was in turn associated with lower IQ scores at 7 years old.

During the child’s hospitalization and after discharge, mothers can present post-traumatic stress disorders (PTSD) symptoms such as avoidance, intrusive thoughts and increased arousal. Rates of PTSD symptoms vary across studies, but are ~15–18% in mothers with perinatal complications (reviewed in Cook *et al*.^28^). The symptoms do not appear to diminish during the first 14 months after the child’s birth^29^, and the frequency is higher still among mothers with premature children that also have many perinatal issues^30^. Previous work likewise would indicate that mothers with emotional distress are less sensitive (i.e., less able to perceive and respond correctly to the child’s signals and needs) to her infant’s signals and is consequently less available to scaffold her child’s development by providing appropriate and reinforcing responses, in turn impacting negatively on the child’s outcomes such as affective and behavioural symptoms, social development and neurodevelopmental outcomes^31–34^.

Links between maternal PTSD symptoms and children’s outcomes beyond infancy remain unresolved and were the focus of the present study. Few studies have considered the severity of perinatal problems, varying for every premature baby, and maternal PTSD symptoms on a preterm-born child’s outcomes^17,28^. Moreover, those that did consider the child’s and mother’s NICU experience investigated early childhood outcomes only^15^. Considering the lack of longitudinal studies and the importance of the transition period to early adolescence (physical and social changes, brain structural reorganization and academic challenges), the purpose of this study was to examine impact of the infant’s perinatal risk factors and the mother’s PTSD symptoms due to premature birth on the child’s intellectual abilities at 11 years old. We expected that high perinatal risk and high numbers of PTSD symptoms would affect negatively a child’s intelligence abilities at early adolescence.

## Results

### Drop- out analyses

Table 1 shows socio-demographic and neonatal data as well as Perinatal Posttraumatic stress Questionnaire (PPQ) scores at 18 months of age for included and dropped out participants. In the full-term (FT) group, results revealed no differences. In the very preterm (VPT) group, results showed a difference in SES scores, the mother’s age at childbirth, birth weight and PErinatal Risk Inventory (PERI) scores. Included participants had higher SES scores, older mothers, higher birth weight, and lower PERI scores than dropped out participants.

**Table 1:** Demographic and perinatal data.

	Included			Dropout			
	1. Full-term	2. Very Preterm	Comparison	3. Full-term	4. Very Preterm	Dropout analysis	
	n = 21	n = 33		n = 10	n = 33		
Socio-demographic	n (%)	n (%)	χ^1–2^	n (%)	n (%)	χ^1–3^	χ^2–4^
Gender (girls)	13 (61.90)	18 (54.50)	0.28	4 (40.00)	12 (36.40)	1.56	2.2
Nationality (Swiss)	17 (81.00)	22 (66.70)	1.83	6 (60.00)	16 (48.50)	4.07	7.13
Parental Status (married)	14 (66.70)	26 (78.90)	0.98	8 (80.00)	29 (87.90)	1.06	3.16
	*M* (*SD*)	*M* (*SD*)	*t* ^*1*– *2*^	*M* (*SD*)	*M* (*SD*)	*t* ^1–3^	*t* ^2–4^
SES	2.91 (0.58)	2.55 (0.53)	2.32*	2.80 (0.97)	2.19 (0.60)	−0.48	−2.59*
Mother’s age at child’s birth (yrs)	32.05 (4.31)	32.18 (4.61)	−0.11	32.10 (4.75)	30.12 (4.57)	0.06	−1.83^†^
Neonatal			U^1–2^			U^1–3^	U^2–4^
Gestional Age (wks)	40.00 (1.29)	30.53 (2.11)	0.00**	40.00 (0.71)	30.32 (2.00)	97.50	496.5
Birth weight (gr)	3305.24 (529.18)	1452.88 (382.85)	0.00**	3272.00 (343.86)	1273.33 (384.89)	101.50	390.50*
Head circumference at birth (cm)	34.56 (0.99)	28.07 (2.39)	0.00**	34.27 (1.97)	27.22 (2.53)	91.50	364.50
PERI	0.19 (0.51)	4.82 (3.04)	6.50**	0.40 (0.84)	6.55 (4.24)	104.50	403.00^†^
			χ^1–2^				
Multiple birth (singletons, n (%))	21 (100.00)	23 (69.70)	7.81*	10 (100.00)	25 (75.80)	—	1.17
Maternal stress at child’s 18 month of age							
	*M* (*SD*)	*M* (*SD*)	*U* ^*1*– *2*^	*M* (*SD*)	*M* (*SD*)	*U* ^1–3^	*U* ^2–4^
PPQ	1.29 (1.62)	4.33 (3.17)	141.00**	1.00 (2.29)	4.12 (3.31)	68.00	505.50

Note. SES: Socio-Economic Status, PERI: Perinatal Risk Inventory score, PPQ: Perinatal Posttraumatic stress Questionare score, χ: Pearson Chi-square, *t*: t of Student, *U*: U of Mann-Whitney. ^†^*p* < 0.1; *p < 0.05; **p < 0.01.

### Socio-demographic, neonatal, and descriptive data

At the child’s birth, VPT participants had lower SES than FT participants. Expected differences between groups were observed for GA, birth weight, head circumference, PERI and PPQ scores. Furthermore, 100% of FT mothers had PPQ scores below clinical threshold (Table 1). At the 11-year-old assessment, results revealed group differences with small effect sizes for age of assessment, IQ and Verbal Comprehension scores (Table 2). The FT group was younger and had higher IQ and Verbal Comprehensive scores than the VPT group. MANCOVA, with SES and child’s age during 11-year assessment as covariates, revealed a trend for a difference between groups (*F*_(1,53)_ = 2.258, *p* < 0.10, η_p_^2^ = 0.125) for IQ score and a reliable group difference for Verbal Comprehension (*F*_(1,53)_ = 5.164, *p* < 0.05, η_p_^2^ = 0.149).

**Table 2:** WISC-IV scores at 11-years assessment.

	Full term	Very Preterm		
	*M (SD*)	*M (SD)*	*t*	*Effect size (η* ^2^ *)*
Age	11.25 (0.17)	11.47 (0.27)	−3.44**	0.19
WISC-IV scores				
Total IQ	114.62 (13.10)	106.00 (14.74)	2.185*	0.08
Verbal Comprehension	119.81 (12.33)	110.03 (13.37)	2.700**	0.12
Perceptual Reasoning	108.10 (16.03)	104.12 (14.95)	0.926	0.02
Working Memory	102.14 (9.16)	96.85 (15.25)	1.594	0.05
Processing Speed	109.67 (13.47)	104.45 (16.54)	1.210	0.03

Note. WISC-IV: Wechsler Intelligence Scale for Children – Fourth Edition, IQ: Intelligence Quotient.

*p < 0.05; **p < 0.01.

### Correlations

Bravais-Pearson bivariate correlations (colloquially referred to in the literature as Pearson correlations) between child’s age at 11-year assessment, SES, PERI, PPQ and IQ and Verbal Comprehension scores were computed (Table 3). After false discovery corrections, results revealed that SES had a marginally significant positive correlation with IQ score. Higher SES scores were associated with higher IQ scores. Moreover, PERI and PPQ scores correlated significantly and negatively with IQ scores. Higher PERI and PPQ scores were associated with lower IQ scores. For Verbal Comprehension scores, a negative correlation was observed with PERI score; higher PERI scores were associated with lower Verbal Comprehension scores. All statistically significant correlations had small effect sizes. Partial correlations, controlling for age at 11-year assessment and SES score, revealed similar results to Bravais-Pearson bivariate correlations, but with smaller effect sizes. Furthermore, Bravais-Pearson bivariate correlations were conducted separately for the FT and VPT groups. After false discovery rate correction, results revealed no significant correlations in either group.

**Table 3:** Bravais-Pearson bivariate correlations and partial correlations.

	Age	SES	PERI	PPQ	IQ	Verbal Comprehension
Age	—	−0.12	0.19	0.17	−0.12	−0.08
SES	—	—	−0.37**	−0.39**	0.28*	0.24^†^
PERI	—	—	—	0.55**	−0.36**	−0.42
PPQ	—	—	0.46**	—	−0.32*	−0.23
IQ	—	—	−0.28*	−0.23	—	0.69**
Verbal Comprehension	—	—	−0.37**	−0.14	0.67**	—

Notes. SES: Socio-Economic Status, PERI: Perinatal Risk Inventory score, PPQ: Perinatal Posttraumatic stress Questionnaire score.

Above the diagonal, Bravais-Pearson coefficient correlation; under the diagonal, partial correlation controlling age and SES score.

^†^*p* < 0.1; *p < 0.05; **p < 0.01.

### Hierarchical regression analysis

**Figure 1:**
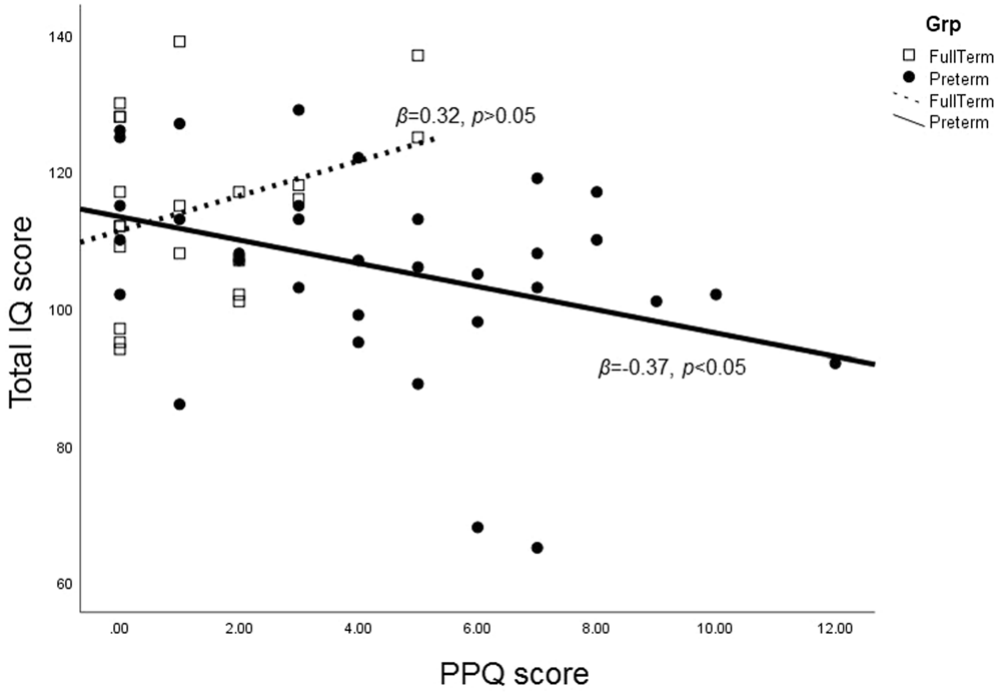
Effect of interaction between groups and PPQ on IQ score.

**Figure 2:**
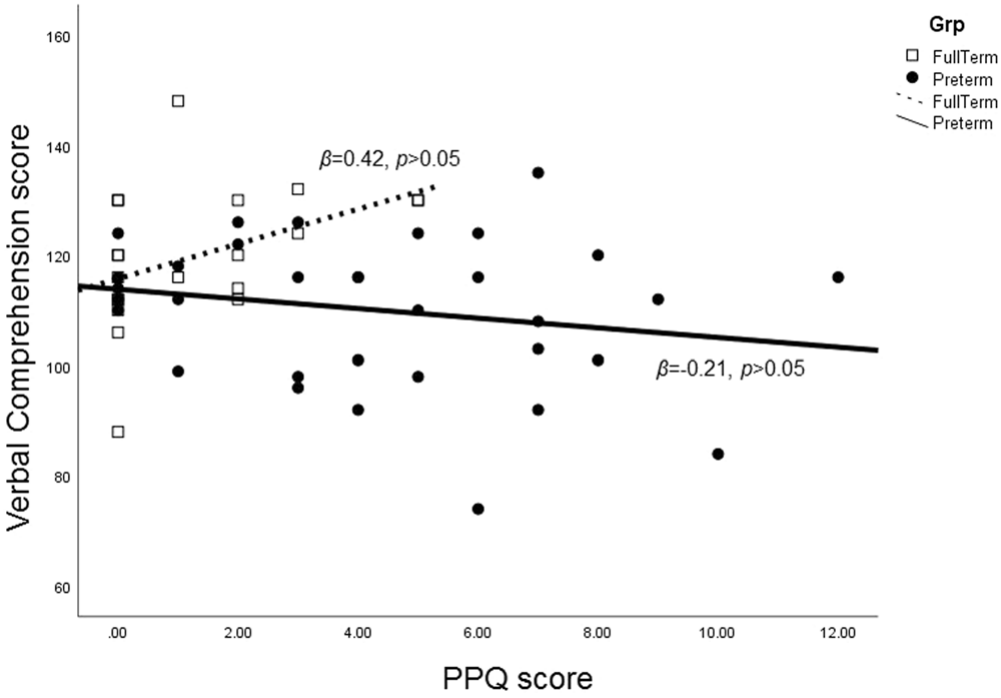
Effect of interaction between groups and PPQ on Verbal Comprehension score.

To study the effect of PERI and PTSD symptoms on IQ at 11 years old, hierarchical regressions were computed. IQ score was significantly explained by the step 3b (F_(6,46)_ = 2.333, p < 0.05, R^2^ = 0.262, R^2^_change_ = 0.24, p < 0.05) of the regression model and in particular by the interaction between PPQ and group (Table 4). In the VPT group, higher PPQ for the mother was linked with a lower IQ score for the child (*β* = −0.365, *p* < 0.05), whereas in the FT group this link was not significant (*β* = 0.315, p > 0.05) (Fig. 1). Likewise, the model of Verbal Comprehension score was significant (*F*_(7, 46)_ = 2.399, *p* < 0.05, *R*^2^ = 0.267, *R*^2^_*change*_ = 0.27, *p* < 0.05). PPQ of the mother could reliably predict the child’s Verbal Comprehension score. The interaction between PPQ and group was also significant (Table 4). However, post-hoc linear regressions releveled no significant effects for either group alone (Fig. 2).

**Table 4:** Hierarchical regression model predicting total IQ score and Verbal Comprehension score at 11 years of age.

*Steps*	*Predictors*	*R* ^2^	*B*	*SE B*	*β*	*t*	*p*
*IQ total*							
step 1	SES	0.09	6.91	3.45	0.27	2.00	0.05
	Child’s age		−4.65	7.68	−0.08	−0.61	0.55
step 2	SES	0.17	3.54	3.74	0.14	0.95	0.35
	Child’s age		−1.44	8.42	−0.03	−0.17	0.87
	Group		−0.63	6.1	−0.02	−0.10	0.92
	PERI		−0.97	0.88	−0.22	−1.11	0.27
	PPQ		−0.63	0.79	−0.13	−0.80	0.43
step 3a	SES	0.17	3.57	3.80	0.14	0.94	0.35
	Child’s age		−1.45	8.51	−0.03	−0.17	0.87
	Group		−0.74	6.41	−0.03	−0.12	0.91
	PERI		−0.96	0.90	−0.22	−1.07	0.29
	PPQ		−0.60	0.91	−0.13	−0.66	0.51
	PPQxPERI		−0.01	0.18	−0.01	−0.06	0.95
step 3b	SES	0.24*	3.97	3.62	0.16	1.1	0.28
	Child’s age		−0.60	8.15	−0.01	−0.07	0.94
	Group		−7.32	6.69	−0.25	−1.10	0.28
	PERI		−0.84	0.85	−0.19	−1.00	0.33
	PPQ		3.00	1.90	0.63	1.58	0.12
	**PPQxGroup**		**−4.23**	**2.03**	**−0.73**	**−2.08**	**0.04**
*Verbal comprehension*							
step 1	SES	0.06	5.66	3.28	0.24	1.73	0.09
	Child’s age		−2.70	7.30	−0.05	−0.37	0.71
step 2	SES	0.20	2.51	3.45	0.10	0.73	0.47
	child’s age		2.45	7.77	0.05	0.31	0.75
	Group		−3.93	5.60	−0.14	−0.70	0.49
	PERI		−1.345	0.811	−0.323	−1.659	0.104
	PPQ		0.24	0.73	0.05	0.33	0.75
step 3a	SES	0.21	2.80	3.48	0.12	0.80	0.43
	Child’s age		2.31	7.80	0.04	0.30	0.77
	Group		−5.28	5.88	−0.19	−0.90	0.37
	PERI		−1.24	0.82	−0.30	−1.51	0.14
	PPQ		0.56	0.84	0.12	0.67	0.51
	PPQxPERI		−0.13	0.16	−0.12	−0.79	0.44
step 3b	SES	0.27*	2.91	3.33	0.12	0.87	0.39
	Child’s age		3.24	7.51	0.06	0.43	0.67
	Group		−10.21	6.16	−0.36	−1.66	0.10
	PERI		−1.22	0.79	−0.29	−1.56	0.13
	**PPQ**		**3.64**	**1.75**	**0.81**	**2.08**	**0.04**
	**PPQxGroup**		**−3.97**	**1.87**	**−0.73**	**−2.12**	**0.04**

Note. SES: Socio-Economic Status, PERI: Perinatal Risk Inventory score, PPQ: Perinatal Postraumatic Questionnaire score, *p < 0.05 for *R*^*2*^ change.

### Receiver operating characteristic (ROC) analysis

**Figure 3:**
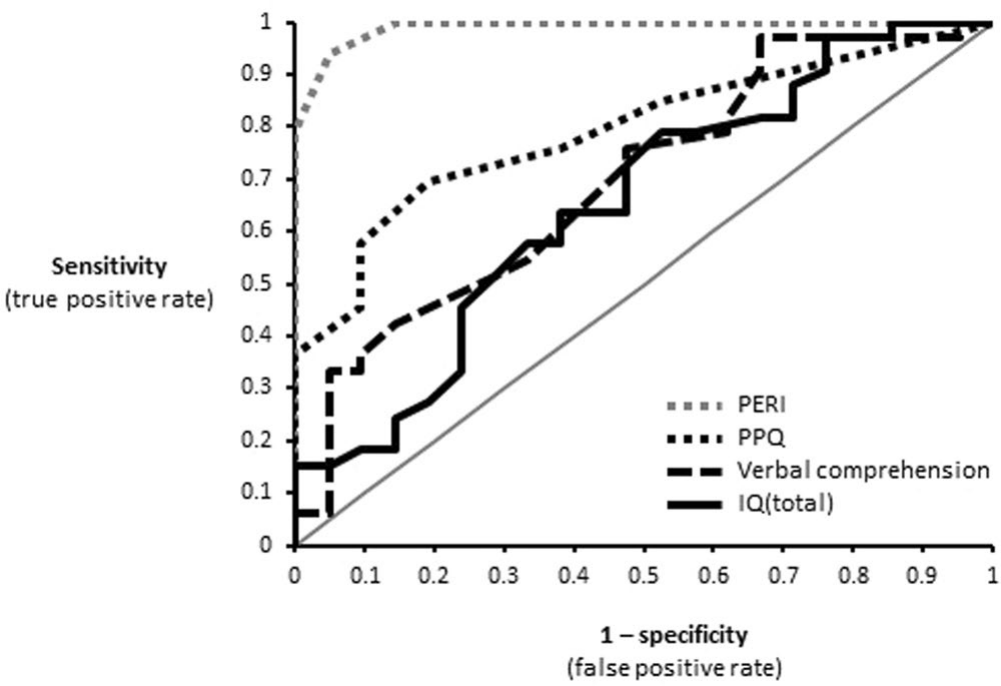
Area under the ROC curve for the classification of FT vs. VPT children. The gray line indicates chance levels. Specific values are detailed in Table 5.

To determine if VPT and FT children could be reliably classified based on the PERI, PPQ, IQ and Verbal Comprehension scores, ROC analyses were performed and evaluated using the area under the curve (AUC) versus a null hypothesis of chance classification. Unsurprisingly, PERI provided nearly perfect classification of VPT from FT children, with an AUC approaching 1 (Table 5). Notably, the child’s IQ score at age 11 as well as the mother’s PTSD symptoms as recalled at 18 months after the child’s birth (i.e. PPQ) also led to reliable classification of the child as VPT versus FT (Table 5 and Fig. 3).

**Table 5:** The area under the curve (AUC) values and their statistical significance for each of the tested variables for the classification of FT vs. VPT children.

*Variable*	*AUC* (±*s*.*e*.*m*.)	*p-value* (*vs*. *H*_0_ *of AUC* = *0*.*50*)	*95*% *Confidence Interval*	
			*Lower bound*	*Upper bound*
PERI	0.99(0.01)	<0.001	0.97	1.00
PPQ	0.79(0.06)	<0.001	0.67	0.91
Verbal Comprehension*	0.70(0.07)	0.02	0.55	0.84
IQ total*	0.65(0.08)	0.06	0.50	0.81

The asterisk indicates that the classification was based on smaller values being predictors of positive state (i.e. classification as VPT).

## Discussion

It is well known that preterm born children have lower IQ scores than their full-term peers^2^. To explain this impairment most researchers focus on GA, birth weight and parental education. Moreover, most prior studies focused on early childhood. The purpose of this study was to consider consequences of child’s perinatal risk and the mother’s PTSD symptoms due to birth on the child’s intelligence abilities at 11 years old. It was hypothesized that both the child’s perinatal risk and the mother’s PTSD symptoms around the time of the birth itself affect negatively the child’s intelligence abilities, extending even into early adolescence. Our principal discovery is that although perinatal factors explain in part preterm born children’s difficulties at birth, maternal emotional distress appears to be a better predictor for intelligence abilities during early adolescence. The child’s perinatal risk itself did not seem to have direct, long-term consequences on the child’s intelligence abilities at 11 years old. However, this may be partially explained by the overlap between the predictors “group” and “PERI”. Differentiating their respective contributions to long-term outcome will surely benefit from continued research. Collectively, the results emphasize the importance of considering not only the preterm born child’s medical situation, but also the mother’s mental well-being in the child’s favorable development.

When SES and age were controlled, the hierarchical regression analysis revealed in the VPT group that maternal PTSD symptoms affected later child’s intelligence abilities, while the child’s perinatal risk does not. More specifically, the more the mother reported PTSD symptoms when the child was 18 months old, the lower was the preterm born child’s IQ scores at 11 years old. In addition, our results show that maternal PTSD symptoms affect negatively child’s verbal competencies independently of whether or not the child was in the FT or VPT group.

These results are in agreement with previous studies revealing poor impact of child’s perinatal complications on intelligence abilities, but still show strong influences of familial factors, such as stability in the family, parenting stress and the quality of the mother-infant relationship^9,15,35,36^. Many studies^33,37,38^ showed the importance of maternal sensitivity and responsiveness in the interactions with their infants to enhance preterm born children’s outcomes. For example, Woodward *et al*. showed that at 4 years old children whose mothers experienced more stress in the NICU have more delays in language development than children whose mothers experienced lower stress^39^. Maternal emotional distress due to premature birth has a deleterious impact on mother-child interactions^40^. More precisely, when compared with mothers of full-term infants, mothers of preterm infants are less sensitive and more controlling in mother-infant interactions, and the controlling pattern is more frequent in mothers with higher PTSD symptoms. Furthermore, compared to children with sensitive mothers, children with controlling mothers present more behavior and affective problems, and lower developmental outcomes^33,37^. The long-term positive effect of sensitive parenting was also demonstrated by Wolke *et al*., revealing that for premature born children, the positive impact of sensitive parenting during middle childhood is particularly evident in school success at 13 years old^36^. This preterm children’s responsiveness to environmental factors is explained by the diathesis-stress model^34,38^. Diathesis stress suggests that considering preterm babies’ physiological immaturity and lower self-regulation; they are more dependent on their environment, in particular the mother’s emotional distress, to regulate their behavior^34^. By revealing the plausible negative influence of maternal PTSD symptoms on premature young adolescents’ intelligence abilities, our results strengthens support of the diathesis-stress model and the importance to consider maternal emotional distress and its effect on mother-infant interaction, in preterm born children development.

Our study had several limitations that we briefly discuss here. First, the PPQ was completed when the child was aged 18 months. This may not exclusively reflect the mother’s PTSD symptoms at the child’s birth and may be less reliable than if the questionnaire had been completed earlier. However, we supposed that reporting mother’s PTSD symptoms at child’s 18 months assess better persistent impressions of the child’s early life environment, rather than solely events linked exclusively to the birth itself. Moreover to provide an index of the stability of this measure, we observed that the PPQ completed when the child was aged 18 months is comparable to the scores when the PPQ was again completed when the child was 11 years old (*r* = 0.72. *p* < 0.001), suggestive of high test-retest reliability. Such notwithstanding, it is important to recognize that the PPQ is completed by the participant and is therefore subjective; though it is based on DSM-IV criteria. Future research incorporating biological markers such as cortisol may be necessary to further substantiate the validity of this measure, though stress hormone levels are not a direct index of PTSD severity nor specificity to motherhood-related stress. Relatedly, our study did not assess directly the impact of child-mother interaction quality on intelligence development. Rather, we defined indirect, but quantifiable metrics. Still, it will be useful for future work to assess which metrics and which specific interactions (both in terms of their quantity and quality) are particularly reflective of maternal PTSD and/or contributing to the child’s IQ. Another limitation is that participants’ IQ scores were higher than usually reported. However, the score difference between VPT and FT we observed is consistent with the published literature^2^. Finally, our results may not be totally representative of preterm born children intelligence development. Indeed, our sample was composed of healthy preterm-born children with few developmental problems. The dropout analysis revealed that in the VPT group, the families who participated at the 11-year-old follow-up had higher SES scores, had older mothers, and had children who presented lower PERI scores than in the dropout group. While not invalidating our discoveries here, this pattern nonetheless suggests that longitudinal studies less mobilize families with lower SES probably for multiple reason that need a careful attention from researchers^41,42^.

Considering the long-term deleterious impact of maternal PTSD symptoms on preterm-born children’s intelligence abilities, detecting early maternal PTSD symptoms is a prime concern. An intervention to reduce PTSD symptoms in preterm-born children’s mothers appears essential to enhance preterm born child’s cognitive development. Borghini *et al*. revealed that early intervention reduces maternal PTSD symptoms at child’s 12 months and enhances mother-child interaction quality^43^. However, early intervention presents benefits in early childhood, but appear to have poor long-term effects^44^. Considering the potential impact of maternal PTSD symptoms, we can suggest a longer intervention with focus on maternal emotional distress more than mother-child interaction.

In conclusion, this study revealed that in healthy preterm children, the child’s perinatal risk does not predict intelligence abilities in early adolescence. However, maternal PTSD symptoms due to premature birth appear to be a factor that can affect negatively later child’s intelligence abilities. The present study is consistent with previous studies concerning childhood and underlines the influence of other variables than the child’s GA or birth weight. However, further studies are needed to confirm these preliminary results. The current study highlights the importance of early detection of maternal PTSD symptoms and by extension interventions that help long-term emotional regulation in mothers and that target mother-child interactions improvement during development.

## Methods

### Procedure

The present study is a part of a longitudinal clinical cohort study. After the child’s birth, the mother was informed about the study and asked to sign a consent form for participation in the research. Socio-demographic and infant’s perinatal risk data were collected during the hospitalization period. At the child’s age of both 18 months and also 11 years, a detailed information letter concerning the study was sent to families. Then, mothers were contacted by phone to fix an assessment in the hospital. At approximately 18 months (FT: *M* = 18.35 months, *SD* = 0.40; VPT: *M* = 18.37 months (corrected age), *SD* = 0.52), the mother filled in questionnaires, and at the 11-year follow-up (FT: *M* = 11.25 y.o., *SD* = 0.17; VPT: *M* = 11.47 y.o., *SD* = 0.27) children participated in an assessment including intellectual abilities. Informed consents were signed by mother (for her child and her participation to the study) at child’s birth and at the 11-year follow-up. All the procedures were performed in accordance with relevant guidelines and regulations and were evaluated and approved by the Vaudois Cantonal Ethics Committee for research in humans.

### Participants

All infants born at 33 weeks or less GA during 1998 and hospitalized in the NICU of the university hospital center were considered for inclusion in the VPT group. Infants with malformation or chromosomal abnormalities and parents with psychiatric illness, drug abuse or without fluency in French were excluded. 105 families were eligible to participate. Among them, 20 refused to participate, 12 infants died, and 4 infants were excluded for developmental problems and visual impairment (i.e. strabismus) at 6 months of age (corrected age). At 18 months of age (corrected age), the remaining 69 children were met. Between 18 months of age and the 11-year follow-up, 32 participants dropped out (e.g. refused to participate or were unreachable). At the 11-year follow-up, three participants were excluded due to missing data, and one child was excluded for neuromotor disability. Finally, 33 children without any severe health problems due to prematurity composed the VPT group.

Healthy full-term infants (GA > 37 weeks) were recruited from the maternity ward of the same university hospital center. Exclusion criteria were difficulties during pregnancy or delivery, a parent with psychiatric illness, drug abuse or lack of fluency in French. Among 36 children, 4 participants dropped out (e.g. refused to participate or were unreachable) between birth and the session at 18 months of age. Then, 10 participants dropped out between the session at 18 months of age and the 11-year follow-up. One participant was excluded for missing data. At the 11-year follow-up, 21 children comprised the FT group.

### Measures

#### Socio-Economic Status (SES)

SES was assessed using an adapted version of the Hollingshead Four Factor Index of Socioeconomic Status^45^. The total score combined parents’ education level and work position. SES score is rated on a 4-point scale; higher score means higher SES.

#### Child’s perinatal problem’s gravity

The gravity of the infant’s perinatal problems was evaluated with the Perinatal Risk Inventory (PERI^46^). The PERI is an 18-item inventory assessing perinatal factors such as Apgar scores, gestational age, infant’s head circumference, electroencephalogram, the duration of ventilation, presence or absence of sepsis and/or meningitis, and presence or absence of congenital infection. Each item range is from 0 to 3 on an ordinal scale. The total score is obtained by summing all items. Higher score means higher perinatal risk. A single nurse from the NICU was trained and then completed the PERI calculation at the infant’s discharge from the NICU based on the infant’s medical file. No data were missing in the PERI form.

#### Maternal PTSD symptoms

The French version of the Perinatal Posttraumatic stress Questionnaire (PPQ^47,48^) was used to assess the mother’s PTSD symptoms due to the child’s birth. The PPQ assesses maternal PTSD symptoms that appeared since the child’s birth and persisted at least one month, during the 6 months after birth. It is a 14-item questionnaire and it is dichotomously scored. The questionnaire is based on DSM-IV^49^ criteria of PTSD. The clinical threshold is fixed at 5 points – score under 6 means no PTSD^48^. The instruction to respondent is to remember if they experimented the described symptoms (i.e., Did you have several bad dreams of giving birth or of your baby’s hospital stay?, Were you more irritable or angry with other than usual?). Mothers filled in the PPQ at the time when the child was 18 months of age.

#### Child’s intelligence abilities

The child’s intelligence abilities were measured at 11 years using the Wechsler Intelligence Scale for Children – Fourth Edition (WISC-IV^50^). The test provides five standardized scores (*M* = 100; *SD* = 15): IQ score - measuring general intellectual ability - and four subscales scores containing verbal comprehension, perceptual reasoning, working memory and processing speed.

### Statistical analyses

First, Shapiro-Wilks’ tests was used to verify if data are normally distributed. To compare socio-demographic data between VPT, FT groups and dropout participants, we used Student’s t-tests for normally-distributed and numeric data, and χ^2^ tests for nominal data. Considering that neonatal data and PPQ scores were not normally distributed, Mann-Whitney’s tests were conducted. Student’s t-tests were also used to compare WISC-IV scores between groups. Considering significant differences between groups in SES scores and child’s age during 11-year assessment, Multivariate Analysis of Covariance (MANCOVA) were performed to compare IQ and WISC-IV’s subscales scores between groups. To observe association between SES, PERI, PPQ and WISC-IV scores, Bravais-Pearson bivariate correlations were quantified. Moreover, partial Bravais-Pearson bivariate correlations controlling SES and child’s age at 11-years assessment were performed. Considering the multiple comparison, false discovery rate corrections were performed on all correlations. To study the effect of PERI and PTSD symptoms on IQ at 11 years old, hierarchical regressions were computed. Due to significant differences between groups in SES scores and child’s age during 11-year assessment, both of them were included as controlling variables in the model’s first step. In the second step, group (FT = 0; VPT = 1), PERI and PPQ scores were included and in the last step, either interaction between group and PPQ, or interaction between PPQ and PERI were introduced separately. Considering the plausible overlapping between group and PERI in this analysis, VIF, tolerance and residual distribution were checked and revealed no issues related to collinearity. Moreover, the achieved power (1-β) was computed with the G*Power software^51^. For a large effect size (i.e., those with clinical relevance, *f*^2^ = 0.35), 54 participants and 6 predictors achieved a power of 0.88 to detect significant results, which is considered to be sufficient^52^. Finally, receiver-operating characteristic (ROC) curves and area under the curve (AUC) analyses were used to determine if PERI, PPQ, IQ and Comprehension Verbal scores can differentiate VPT and FT children, and if IQ score can differentiate mothers with or without PTSD. It was unnecessary here to apply a procedure to control for missing data because participants with missing data were removed from analyses. Any Statistical analyses were conducted with SPSS for Windows (version 23.0 Armonk, NY: IBM Corp).

## Data Availability

The datasets generated during and/or analysed during the current study are available from the corresponding author upon reasonable request.
